# New insights into the pharmacokinetics and pharmacodynamics of natalizumab treatment for patients with multiple sclerosis, obtained from clinical and in vitro studies

**DOI:** 10.1186/s12974-016-0635-2

**Published:** 2016-06-27

**Authors:** T. Sehr, U. Proschmann, K. Thomas, M. Marggraf, E. Straube, H. Reichmann, A. Chan, T. Ziemssen

**Affiliations:** Neuroimmunological Lab, Center of Clinical Neuroscience, Neurological Clinic, University Hospital Carl-Gustav Carus, Dresden University of Technology, Fetscherstraße 74, D-01307 Dresden, Germany; Multiple Sclerosis Center, Center of Clinical Neuroscience, Department of Neurology, University Hospital Carl-Gustav Carus, Dresden University of Technology, Fetscherstraße 74, D-01307 Dresden, Germany; Neurology Outpatient Center Barsinghausen, Marktstrasse 27/29, Barsinghausen, 30890 Germany; Department of Neurology, University Hospital Bern and University of Bern, Freiburgstrasse, Bern, 3010 Switzerland

**Keywords:** Multiple sclerosis, Treatment monitoring, Tysabri, Natalizumab, CSF, Immunology

## Abstract

**Background:**

The monoclonal antibody natalizumab (NAT) inhibits the migration of lymphocytes throughout the blood–brain barrier by blocking very late antigen (VLA)-4 interactions, thereby reducing inflammatory central nervous system (CNS) activity in patients with multiple sclerosis (MS). We evaluated the effects of different NAT treatment regimens.

**Methods:**

We developed and optimised a NAT assay to measure free NAT, cell-bound NAT and VLA-4 expression levels in blood and cerebrospinal fluid (CSF) of patients using standard and prolonged treatment intervals and after the cessation of therapy.

**Results:**

In paired CSF and blood samples of NAT-treated MS patients, NAT concentrations in CSF were approximately 100-fold lower than those in serum. Cell-bound NAT and mean VLA-4 expression levels in CSF were comparable with those in blood. After the cessation of therapy, the kinetics of free NAT, cell-bound NAT and VLA-4 expression levels differed. Prolonged intervals greater than 4 weeks between infusions caused a gradual reduction of free and cell-bound NAT concentrations. Sera from patients with and without NAT-neutralising antibodies could be identified in a blinded assessment. The NAT-neutralising antibodies removed NAT from the cell surface in vivo and in vitro. Intercellular NAT exchange was detected in vitro.

**Conclusions:**

Incorporating assays to measure free and cell-bound NAT into clinical practice can help to determine the optimal individual NAT dosing regimen for patients with MS.

**Electronic supplementary material:**

The online version of this article (doi:10.1186/s12974-016-0635-2) contains supplementary material, which is available to authorized users.

## Background

Natalizumab (NAT, Biogen Idec) is a monoclonal IgG4 antibody approved for the treatment of highly active relapsing remitting multiple sclerosis (MS) [1]. Blockade of the interaction between very late antigen (VLA)-4 and vascular cell adhesion molecule (VCAM)-1 at the blood–brain barrier (BBB) reduces the transmigration of leukocytes into the brain, which reduces inflammatory lesions in MS patients [2]. It also has a negative impact on central nervous system (CNS) immunosurveillance, which is responsible for development of progressive multifocal leukoencephalopathy (PML) [3, 4].

The pharmacokinetics and pharmacodynamics of free NAT and the immunological effects of VLA-4 blockade on peripheral immune cells are well known [5–9], but there is little information on the pharmacology beyond the BBB [10]. In addition, only limited information is available about the pharmacokinetics and pharmacodynamics of NAT metabolism after patients stop treatment or if they are treated with NAT infusion intervals longer than 4 weeks [8, 11, 12]. These questions are starting to be explored, and treatment strategies that adjust the NAT dose to the patient’s body weight are being adopted [13]. There also is interest to determine NAT pharmacokinetics and pharmacodynamics in patients with MS who switch to other treatments [14].

To quantify free and cell-bound NAT peripheral blood and CSF, we developed a new immunological assay using fluorescence-activated cell sorting (FACS) analysis. Here, we applied this new assay to in vitro experiments on the exchange of cell-bound NAT with NAT-neutralising antibodies (NABs) and to monitor different clinical scenarios. We determined the NAT characteristics in patients stopping NAT treatment and patients with different infusion intervals. The effects of NAT-neutralising antibodies were investigated using 30 blinded sera of NAT-treated patients.

## Methods

### Subjects

To analyse free and cell-bound NAT, paired CSF and blood samples of 27 MS patients receiving monthly NAT treatment and 24 untreated MS patients were obtained (Table 1*A*). All patients were prospectively documented in the NAT module of MSDS software [15] and were stable for at least 12 months before the study. A total of 13 patients receiving NAT therapy were reevaluated after 12 monthly NAT infusions. No NAT-treated patient exhibited clinical disease symptoms. One patient who was positive for NAT-neutralising antibodies was analysed separately. Blood samples were obtained immediately before and 20 min after NAT infusion, whereas CSF was only obtained immediately before NAT infusion. To evaluate the effects of different infusion intervals on free NAT concentration, we obtained sera of 46 MS patients that were stable for more than 6 months on the following NAT infusion intervals: 4 weeks (*n* = 18), 5 weeks (*n* = 18), and 8 weeks (*n* = 10). CSF was collected only from patients who volunteered for a lumbar puncture (Table 1*B*). To analyse the effect of NAT cessation, blood samples of 18 MS patients who received a mean of 34.8 ± 16.1 NAT infusions were obtained 20 min after their last NAT infusion and monthly during the NAT washout period for up to 4 months (Table 1*C*). Patients were monitored for occurrence of confirmed clinical relapses and of Gadolinium enhancing (GdE) or new T2 lesions in magnetic resonance imaging (MRI). The study procedure was performed according the Declaration of Helsinki and the study protocol was approved by the Ethics Committee of the Faculty of Medicine of the Dresden University of Technology. All participants provided informed written consent.

**Table 1:** Subject characteristics

	(A) Standard treatment interval		(B) Extended treatment intervals			(C) Cessation
	NAT	Non-NAT	4 weeks	5 weeks	8 weeks	
Number of patients	27	24	18	18	10	18
Women (%)	60	70	50	72	67	61
Age in years (mean ± SD)	35.4 ± 9.4	38.5 ± 12.1	36.9 ± 9.4	44.8 ± 6.7	42.7 ± 8.5	38.3 ± 10.2
Years of disease (mean ± SD)	8.9 ± 6.8	1.4 ± 4.6	8.9 ± 6.8	14.5 ± 7.5	10.0 ± 3.0	10.4 ± 8.3
EDSS^a^ (mean ± SD)	3.5 ± 1.6	2.5 ± 1.6	3.5 ± 1.6	3.7 ± 1.1	3.3 ± 1.53	3.7 ± 1.9

^a^EDSS, Kurtzke Expanded Disability Status Scale

### Methods

To measure free NAT concentration, serum samples (dilution 1:100 to 1:750), CSF samples (undiluted) and standard solutions with different NAT concentrations (between 10^−4^ and 10 μg/ml) were added to 96-well plates containing HL60 cells. Cells were stained with anti-IgG4 antibody (Southern Biotech). Mean fluorescence intensity (MFI) was analysed using FACS (FACS Calibur, BD Biosciences). A NAT standard curve was generated in triplicate based on the MFI of standard concentrations (Additional file 1: Figure S1). The minimum calculated detection limit was 14 ng/ml. Intra-assay and inter-assay reliability was consistently below 5 and 10 %, respectively. Antibody specificity was tested using an anti-NAT-neutralising antibody. For NAT saturation and VLA-4 expression, cells were stained ex vivo using CD45 (Dako) and CD3 primary antibodies and anti-CD49d (BD Biosciences) and anti-IgG4 secondary antibodies, respectively. Positive (in vitro maximum-loaded frozen samples), negative (untreated frozen cells) and anti-IgG3 (Southern Biotech) isotype controls were co-analysed. Label saturation was defined as the MFI difference between cells labelled with the anti-IgG4 and anti-IgG3 isotypes.

Cells were analysed as follows. CSF samples were centrifuged within 20 min after collection. Peripheral blood mononuclear cells were isolated from the heparinised blood samples using Biocoll separating solution (Biochrom Ag) and Ficoll-Paque (Amersham Biosciences) in LeucoSep tubes (Greiner Bio One). Plasma and serum supernatants were collected and frozen for subsequent NAT concentration analysis.

To evaluate NAT-dependent VCAM binding, specific dilutions of NAT or biological material (serum and CSF) were added to HL60 cells. Then, VCAM-IgG1 His-Tag Chimera (R&D Systems) was added to cells. Cells were stained with anti-IgG4 and anti-His-Tag (Lifespan Biosciences) antibodies and then analysed by FACS.

For in vitro analysis of intercellular NAT exchange, NAT-loaded and non-loaded carboxyfluorescein succinimidyl ester (CSFE)-marked HL60 cells were cocultured for 24 h at 37 °C either mixed at 1:1 ratio or separated by a permeable membrane (pore size 0.4 μm, Millipore Transwell). Then, HL60 cells were analysed for IgG4 MFI mean by FACS and compared with separately cultured control cells.

A blinded analysis was performed using 30 sera of NAT-treated patients with or without NABs (provided by Dr. Gold and Dr. Chan, from the German NABs laboratory in Bochum, Germany). Different sample dilutions of sera were added to the HL60 cells, and the resulting MFI mean of cell-bound NAT was analysed by FACS. In a second experiment, dilutions of serum samples containing NABs were added to standardised NAT concentrations (used to generate the standard curve). The resulting MFI mean of anti-IgG4 binding was compared with that of the standard curve.

To investigate the effect of NABs on cell-bound NAT in vitro, maximally NAT-loaded HL60 cells were incubated with different dilutions of serum containing NABs for different time intervals. Then, cell-based and free NAT were measured in the supernatant. Data are expressed as mean values with standard error of the mean or standard deviation. Correlations were analysed using Spearman’s rank correlation coefficients. Wilcoxon’s matched pair test was used to analyse pre-infusion and post-infusion differences or other paired analyses. The unpaired *t* test or the Mann–Whitney test was used for other group comparisons. *p* < 0.05 was considered as statistically significant. All statistical analyses were performed using Graph Pad Prism 5.

## Results

### Free NAT levels are 100-fold lower in CSF relative to serum

**Fig. 1:**
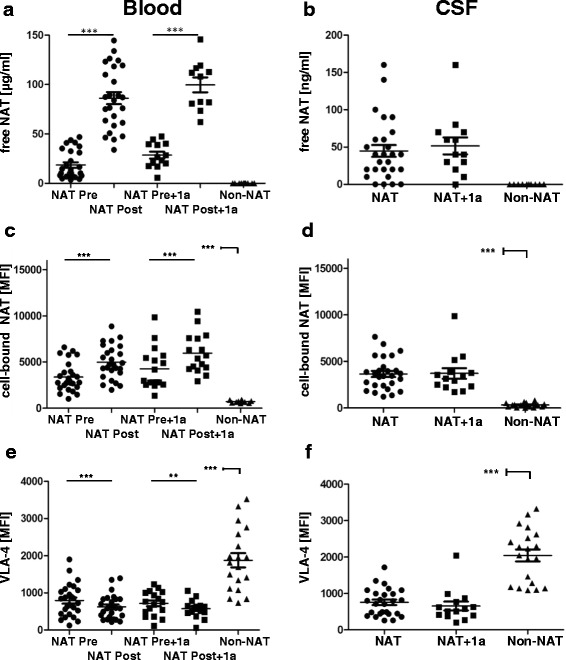
Free NAT, cell-bound NAT and VLA-4 expression levels in regular NAT-treated patients. Free NAT concentration (**a**, **b**), cell-bound NAT on CD3 cells (**c**,**d**) and VLA-4 expression level in CD3 cells (**e**,**f**) of NAT-treated (NAT; *black circle*) and untreated (non-NAT; *black triangle*) patients with MS were determined in blood (left) and CSF (right). One year later (after 12 more NAT infusions), 13 NAT-treated patients with MS were re-analysed (NAT + 1a; *black square*). NAT values in blood were determined before (NAT Pre) and 20 min after (NAT Post) NAT infusion. **p < 0.01, ***p < 0.001

Mean free NAT serum concentration was 18.5 μg/ml (SD ± 15.3) before NAT infusion and 86.3 μg/ml (SD ± 31.3) immediately after NAT infusion, which represents an increase of approximately 4.7 times (Fig. 1a). For CSF, the mean free NAT concentration in CSF was 44.8 ng/ml (SD ± 41.4) (Fig. 1b). Although there were high inter-individual variabilities, patients with high NAT levels pre- and post-infusion at time point one displayed comparable pre- and post-infusion levels at time point two (after having received 12 more NAT infusions) with a correlation coefficient of *r* = 0.86 (*p* < 0.0001) (Additional file 2: Figure S3). No significant differences were observed in mean free NAT concentration between the two time points. During the first and second years, no free NAT was detected in the CSF or serum of 4/27 and 1/13 patients, respectively, and in all CSF specimen of the untreated control group.

### Free and cell-bound NAT in blood and CSF show different kinetics in blood and in CSF

The relative MFI of NAT on the cell surface before NAT infusion was comparable in CSF (3.653, SD ± 1700) and blood (3.375, SD ± 1545) for all NAT-treated patients (Fig. 1c, d), whereas untreated controls had low MFI in CSF and blood (322 and 710). The MFI after one NAT infusion increased approximately 1.4 times, which was lower than the observed increase in free NAT. We did not detect any significant differences in these values during 1 year of treatment.

### VLA-4 expression is reciprocally regulated in blood and CSF, compared to cell-bound NAT

The relative VLA-4 amount on the cell surface before NAT infusion was comparable in CSF (756, SD ± 376) and blood (794, SD ± 426) for all NAT-treated patients (Fig. 1e, f). The VLA-4 MFI after a single NAT infusion was approximately 1.3 times lower than that before infusion. Controls showed much higher VLA-4 expression levels. The CSF-to-serum ratios of albumin and IgG concentrations were significantly correlated (*r* = 0.74, *p* < 0.0001) with the CSF-to-serum ratio of free NAT (*r* = 0.44, *p* < 0.057), suggesting that the free NAT distribution was dependent on free diffusion and blood–brain barrier damage (such as albumin or IgG). We found that free and cell-bound NAT in blood and CSF were significantly correlated (*r* = 0.49, *p* < 0.0014 and *r* = 0.47, *p* < 0.023), which likely represents a steady state between free and cell-bound NAT (Additional file 3: Figure S2). One patient with NAT-neutralising antibodies had similar values (free and cell-bound NAT and VLA-4 expression) as those of the untreated control patients (data not shown).

### Free and cell-bound NAT as well as VLA-4 in patients after NAT cessation and different infusion intervals

**Fig. 2:**
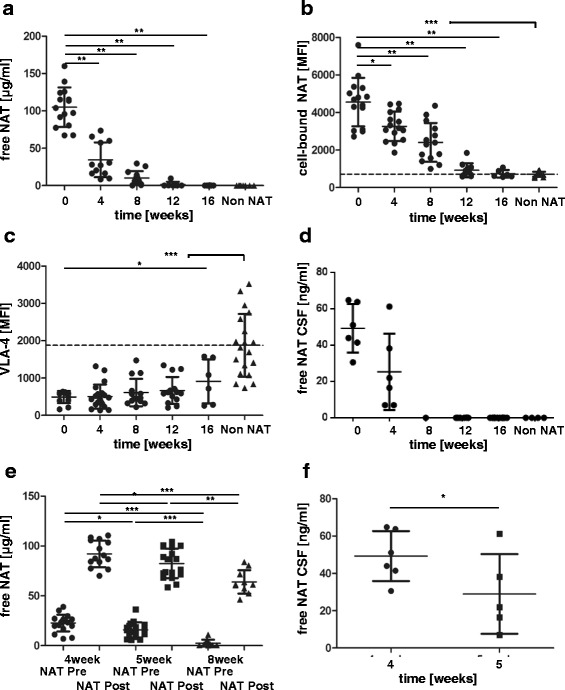
Free NAT, cell-bound NAT and VLA 4 expression levels in patients subjected to extended treatment intervals and after cessation of therapy. Free NAT concentration (**a**), cell-bound NAT on CD3 cells (**b**) and VLA-4 expression in CD3 cells (**c**) in blood after cessation of NAT treatment (NAT; *black circle*) compared with that in untreated patients with MS (non-NAT; *black triangle*). Values were determined 20 min after the last NAT infusion, at baseline, and for different patients during the subsequent 16 weeks. Free NAT concentration in CSF after cessation of NAT therapy (**d**). Free NAT concentrations of patients receiving 4- (*black circle*), 5- (*black square*) and 8-week (*black triangle*) intervals between treatments (**e**). Free NAT concentration in CSF of patients receiving 4- (black circle) and 5-weeks (black square) intervals between treatments (**f**). Blood values were determined before (NAT Pre) and 20 min after (NAT Post) NAT infusion. *p < 0.1, **p < 0.01, ***p < 0.001

After NAT cessation, we measured exponential decreases of free NAT levels in the peripheral compartment of 67.2, 90.3, 98.6 and 99.9 % from weeks 4, 8, 12 and 16 after the last drug administration compared to baseline NAT levels in the same patients (Fig. 2a). NAT was detectable in 6 of 7 patients after 12 weeks after the last infusion, and in 7 of 9 patients 16 weeks after the last infusion. Cell-bound NAT decreased by 33.6, 55.9, 94.4 and 99.3 % from weeks 4, 8, 12 and 16 after last infusion compared with baseline levels in the same patients compared to the more gradual decline in free NAT levels in the same patients (Fig. 2b). By 12 weeks after the last infusion, no significant difference in IgG4 binding was detected compared with that of the control group. VLA-4 expression displayed a slow increase after NAT cessation of 2.8, 24.2, 35.5 and 85.5 % by 4, 8, 12 and 16 weeks after the last infusion. At 16 weeks, VLA-4 expression was less than 50 % of that of the control group (Fig. 2c).

Reductions in cell-bound and free NAT and increases in VLA-4 expression are clinically linked to the recurrence of disease activity. One third of the patients presented clinically confirmed relapses whereas nearly two thirds showed new lesions in MRI (80 % GdE lesions). We still detected very low NAT concentrations in CSF samples months after the cessation of NAT treatment (Fig. 2d). In treatment groups that received NAT infusions every 4, 5 or 8 weeks, significantly higher mean free NAT concentrations were detected in patients with 4-week intervals (22.69 ± 8.37 μg/ml) than in patients with 5-week (15.84 ± 7.58 μg/ml) and 8-week (2.49 ± 0.46 μg/ml) intervals (Fig. 2e). After infusion, the NAT concentration was significantly lower in the 8-week interval group (63.95 ± 11.75 μg/ml) than in the 4-week (92.11 ± 13.3 μg/ml) and 5-week (82.36 ± 14.72 μg/ml) interval groups (Fig. 2e). These results suggest a higher binding capacity for free NAT after longer intervals between infusions. In the CNS compartment, the NAT concentration was significantly higher in the 4-week (49.26 ± 13.36 ng/ml) than in the 5-week (28.97 ± 21.95 ng/ml) treatment interval groups (Fig. 2f). None of the patients in any of the three treatment groups displayed any clinical activity of MS disease symptoms during the investigated time period.

### Functional assay of VCAM-1 binding effects in vitro

**Fig. 3:**
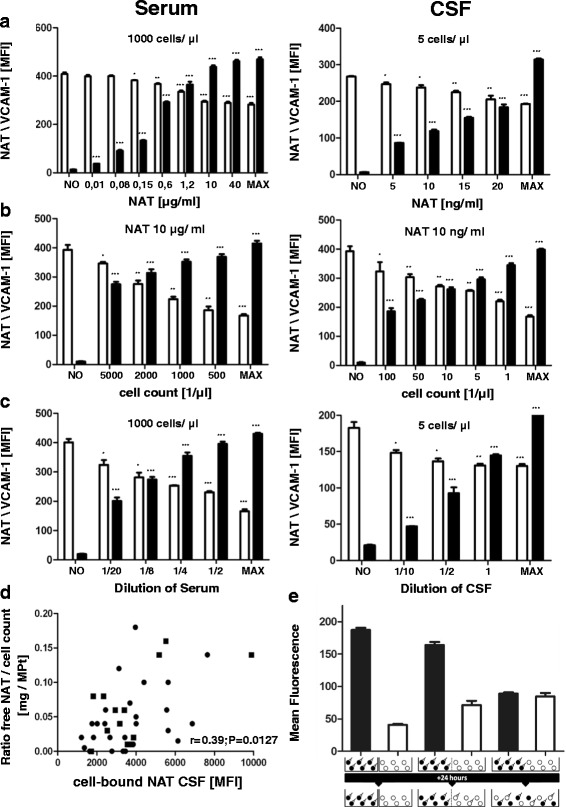
Functional assay of VCAM-1-binding effects in vitro. **a** Cell-bound NAT (black bars) and VCAM-1 (white bars) MFI values after incubation of different numbers of cells comparable to those in vivo in blood or serum (5 or 1000/μl, respectively) with and without (NO) different NAT concentrations, up to maximally loaded cells [MAX] with 100 % NAT saturation. **b** Incubation of different numbers of cells with NAT concentrations comparable to those in vivo in blood or serum (10 ng/ml and 10 μg/ml, respectively). **c** Incubation of 5 or 1000 cells/μl with different dilutions of CSF or serum of NAT-treated patients with MS. *p < 0.05, **p < 0.01, ***p < 0.001. **d** Correlation of cell-bound NAT with the ratio of free NAT concentration-to-cell count in CSF. Values from NAT-treated patients with MS (*black circle*) who were partly reevaluated after 1 year (*black square*). **e** Mean fluorescence intensity (MFI) of cell-bound NAT after (from *left* to *right*) separation, co-cultivation but separated by a permeable membrane, or free co-cultivation of NAT-preloaded (black bars) and non-loaded (white bars) CSFE-marked cells for 24 h. The conditions and results are illustrated below the figure

To understand the relationship between free and cell-bound NAT and the lymphocyte count in blood and CSF, we performed in vitro experiments and added different NAT concentrations to specific numbers of cells to simulate the cellular concentration in CSF or blood compartment (5 or 1000 cells/μl, respectively). To evaluate the functional VLA-4 activity blocked by NAT, we measured VCAM-1 binding activity and cell-bound NAT. The addition of increasing NAT concentrations caused a dose-dependent decrease of functional VCAM-1-binding (Fig. 3a). To confirm this result, we used different cell numbers with constant NAT concentrations and observed an increase of cell-bound NAT and a decrease of VCAM-1-binding with decreasing cell quantities (Fig. 3b). CSF and serum samples of several NAT-treated patients were tested, and functionally active NAT was detected ex vivo illustrated by decreasing VCAM-1 binding (Fig. 3c). We detected maximally loaded cells in vitro using cell counts similar to those in vivo in CSF and blood.

There was a significant correlation between cell-bound NAT and the ratio of free NAT concentration in CSF and the cell count (Fig. 3d). To understand potential exchange between the different NAT pools, we performed co-cultivation experiments using NAT-loaded, CSFE-labelled cells cultivated with unloaded and unlabelled cells for 24 h (Fig. 3e). The results showed that there was a quick and complete exchange of cell-bound NAT between labelled and unlabelled cells. Then, we separated both cell groups by a NAT-permeable membrane, and still detected significant but incomplete NAT exchange. These results indicate that cell-cell contact enables quick and complete NAT exchange, whereas only limited exchange of NAT occurs without cell-cell contact. Cell-bound NAT appears to serve as a NAT reservoir.

### NAT-neutralising antibodies remove cell-bound NAT in vitro

**Fig. 4:**
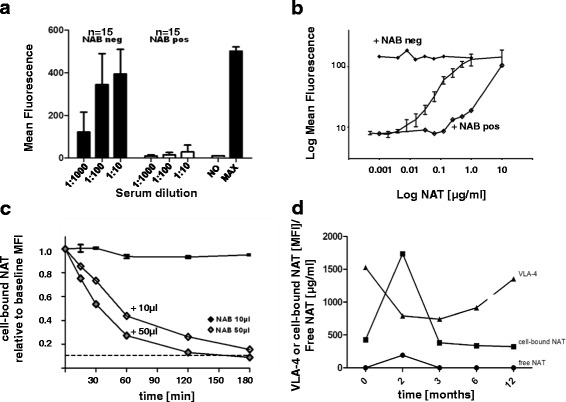
Analysis of NAT-neutralising antibody (NAB) in vitro. **a** Cell-bound NAT mean fluorescence intensity (MFI) on HL60 cells after adding different dilutions of blinded NAB-positive or NAB-negative serum samples. MFI values were compared with those of non-loaded (NO) and maximally loaded (MAX) control cells. **b** Example standard curves (*solid line* with *error bars*) after adding NAB-positive (NAB pos) or NAB-negative (NAB neg) serum samples. **c** Cell-bound NAT decreases (relative to baseline MFI) over time after the addition of control solution without NAB (*solid line* with *error bars*) or different NAB volumes [10 μl and 50 μl]. *Dotted line* represents the MFI of non-loaded cells. **d** Free NAT, cell-bound NAT and VLA-4 expression levels in a patient over infusion time who was tested positive for NAB during NAT infusion therapy

The cellular effects of NAT-neutralising antibodies were evaluated in blinded experiments by incubating HL60 cells with serum samples from patients that were positive or negative for NAT antibody (15 serum samples for each). In these blinded experiments, all different samples were correctly identified as either NAB-positive or NAB-negative cell reactions. First, in the presence of serum containing NAT-neutralising antibodies, no increase in cell-bound NAT was detected (Fig. 4a). Second, adding different dilutions of serum containing NAT-neutralising antibodies to HL60 cells incubated with standard NAT dilutions causes a release of cell-bound NAT from the cells. We measured a reduction in the MFI means of anti-IgG4 binding, and the standard curve was shifted to different degrees (Fig. 4b).

The next in vitro experiment showed that NAT-neutralising antibodies neutralised or released cell-bound NAT. We incubated maximally NAT-loaded HL60 cells with serum containing NAT-neutralising antibodies to simulate in vivo NAT concentrations with respect to cell counts. The results showed that there was a decrease in cell-bound NAT within the first minutes and a complete loss within 3 h. This effect was dose-dependent (Fig. 4c). The removed NAT appeared to form a complex with the NAT-neutralising antibodies, as our assay did not detect free NAT in the supernatants. Longitudinal analysis of a patient who developed NAT-neutralising antibodies showed the characteristic effects on VLA-4, free NAT and cell-bound NAT. Before the occurrence of NAT-neutralising antibodies, increases in free and cell-bound NAT and a decrease in VLA-4 expression were detected after starting NAT treatment. After developing NAT-neutralising antibodies, the opposite effects were observed (decreases in free and cell-bound NAT and an increase in VLA-4 expression). These reaction parameters could be used as markers for in vivo monitoring of NAT-neutralising antibodies in patients with MS (Fig. 4d).

## Discussion

Quantification of free NAT, cell-bound NAT and VLA-4 expression levels may provide a better understanding of the underlying mechanisms and management of NAT therapy. In this study, we developed a highly sensitive FACS assay to measure NAT concentrations in CSF. We evaluated the assay using a cell line that has high expression of VLA-4, anti-IgG4 antibodies and a specific, highly active anti-NAT-neutralising antibody and showed that the assay had low inter-assay and intra-assay variability for quantification of free and cell-based NAT in CSF and blood.

We found that NAT was detectable in the CSF of patients who received long-term NAT therapy, which was consistent with a recent report [10]. In contrast with that trial, we only included patients who received NAT infusions every 4 weeks, and we determined CSF and serum NAT levels immediately before NAT infusion, which may explain the differences in measured NAT levels in the former and current studies. Harrer et al. reported that the NAT concentration in the CSF was more than 400 times lower than that in serum [10]. This result is consistent with the concept of the CSF as an ultrafiltrate of plasma, which allows proteins with a hydrodynamic radius smaller than 500 nm to penetrate the CNS compartment. The NAT concentrations may be higher in patients with damage to the BBB. We found a close correlation between the CSF-to-serum ratios of NAT, albumin and IgG. However, we did not detect any correlation between infusion number and NAT concentration in our patient cohort, and there were no significant differences between serum and CSF NAT concentrations in a subgroup of NAT-treated patients that was analysed 1 year later. We detected large inter-individual variability of NAT levels in serum and CSF, similar with previous reports [10, 16–18].

We also found that there was a much more pronounced decline in free NAT levels during a 4-week interval than cell-bound NAT.

The presence of NAT in the CNS immune compartment may have important implications because the functional properties of T cells that migrate to the CNS could be modulated in the same way as previously described for peripheral blood [19–21]. Our in vitro and ex vivo experiments indicate that CSF-localised NAT is functional. Although NAT is mainly active by inhibiting migration via the blood–brain barrier [22], there is potential role for functional NAT in the CSF compartment as T cells are still present in the CSF [23]. In contrast to Schneider-Hohendorf et al. who described the lack of VLA-4 of CSF T cells in NAT-treated patients [24] we could clearly demonstrate cell-bound NAT on the surface of the remaining CSF T cells using our assay to detect cell-bound NAT. The binding of VLA-4 to the sVCAM binding was blocked by NAT binding to VLA-4 [25]. The possible VLA-4 blockade of CSF T cells by functional NAT in the CSF may be relevant for inhibiting their further intraparenchymal migration to the inflammatory lesion site as VLA-4 mediates cell-extracellular matrix interaction via, e.g., fibronectin or osteopontin [26, 27] and may perpetuate the inflammatory cascade within the brain parenchyma [28]. The VLA-4-fibronectin interaction also acts as a costimulatory or second signal (in addition to T cell receptor signalling) for lymphocyte proliferation and activation [29]. Although NAT concentration in the CSF was much lower than those in blood, the amount of NAT that is bound to the cell surface was comparable in blood and CSF due to the lower cell count in CSF.

We investigated different NAT-dosing intervals in addition to the standard interval of 4 weeks. All patients continuously treated with infusion intervals of 5 or 8 weeks were stable on clinical and MRI monitoring. However, NAT concentration continuously declined in CSF and serum when the NAT-dosing interval was longer than 8 weeks. Combining free and cell-bound NAT assessments, our assay may provide a unique tool for the analysis of individual NAT-dosing regimens to identify the lowest NAT dose that still provides optimal efficacy. Prior studies have also identified a relationship between patient weight and NAT. A decrease in dose adapted to the pharmacodynamics effect of NAT may lower the PML risk in NAT-treated patients as it has been proposed for two recently described clinical cohorts on prolonged NAT-dosing intervals [30, 31]. The number of patients included in this study was not high enough to individually link clinical efficacy and free respectively cell-bound NAT concentration. However, we observed more pronounced differences between pre- and post-dosing NAT concentrations with longer infusion intervals, suggesting that individualised doses applied every 4 weeks may have greater benefits of a constant and dose-adapted pharmacodynamic effect of VLA-4 blockade than longer individual infusion intervals with a fixed dose.

Monitoring the cessation of NAT treatment revealed different kinetics of free and cell-bound NAT. Free NAT levels decline rapidly, whereas the pharmacodynamically relevant changes of cell-bound NAT and VLA-4 expression take significant longer time to return to baseline. The link between pharmacodynamics and the return of disease activity has already been previously described [32–34]. Elimination of monoclonal antibodies is generally slow and involves mechanisms that differ among patients. Rispens et al. reported that rebound disease activity occurred later in patients who showed the highest NAT levels [18]. Another important clinical argument for measuring free and cell-bound NAT, especially in CSF, is the determination of when to start alternative treatments in patients switching from NAT [35].

Our in vitro experiments demonstrated that there is a quick cell-to-cell exchange of NAT antibody. Ex vivo and in vitro results showed that cell-bound NAT can serve as a NAT reservoir. In in vitro experiments, free NAT was not detectable until all cells were maximally loaded. Our blinded experiment could correctly identify all NAB-positive and NAB-negative patients. In contrast to the results of Pilz et al. [36], we could detect significant effects of NAT loading in all of our NAB-positive patients. Our analysis of NAB-positive patients indicated that presence of NAB reduced free NAT levels and was even able to remove cell-bound NAT from the VLA-4 receptor. In addition, the area under curve serves as a quantitative functional estimate of the titer and potency of NAT-neutralising antibodies as differences regarding the magnitude of the effect could be described for individual patients. Up to now, only enzyme-linked immunosorbent assay (ELISA) and radioimmunoassay (RIA) analysis is available for the detection of NAB which cannot describe the functional effects of NAB in comparison to our assay [37–39]. Further analysis and improvements are necessary to develop our test for the evaluation of NAB titer (quantity of NAB) and potency (ability to remove cell-bound NAT).

This effect of NAT removal by NAB occurred in a dose-dependent manner within a few hours. Such effective NAB could theoretically be used to remove NAT from the cell surface if, for example, PML would have been diagnosed. This would be a much more effective and faster alternative to plasma exchange which can accelerate NAT clearance only by an average of 92 % within 1 week [7].

## Conclusions

Our results suggest that it is important to include free and cell-bound NAT concentrations in addition to VLA-4 saturation and functional migration assays in future studies to link immunology and clinical outcomes for NAT treatment. Cell-bound NAT and VLA-4 saturation could be important surrogate markers for NAT efficacy to identify optimal doses for each patient [8, 40]. These parameters could be used to optimise an individual dosing regimen and determine the best time to start an alternative treatment after the cessation of NAT therapy.

## Abbreviations

BBB, blood brain barrier; CNS, central nervous system; CSF, cerebrospinal fluid; CSFE, carboxyfluorescein succinimidyl ester; ELISA, enzyme-linked immunosorbent assay; FACS, fluorescence activated cell sorting; GdE, gadolinium enhancing; MFI, mean fluorescence intensity; MRI, magnetic resonance imaging; MS, multiple sclerosis; NABs, natalizumab neutralising antibodies; NAT, natalizumab; PML, progressive multifocal leukoencephalopathy; RIA, radioimmunoassay; VCAM-1, vascular cell adhesion molecule; VLA-4, very late antigen-4

## Additional files

Additional file 1: Figure S1. Standard curve of NAT assay. Specific numbers of HL60 cells were incubated with defined NAT concentrations and produced repeatable mean fluorescence intensity (MFI). These values were used to create a standard curve for NAT concentration (connected line). Specificity was tested using NAT-neutralising antibodies of a NAT-treated patient with multiple sclerosis (resulting value white circle). (PPTX 112 kb) Additional file 2: Figure S3. Correlation of pre- and post-serum values at both timepoints. Correlation of pre- (black circle) and post- (white circle) NAT serum values at timepoint 0 and after 1 year (+1a). *r* = Spearman’s rank correlation coefficient, *p* = *p* value of significance. (PPTX 45 kb) Additional file 3: Figure S2. Correlation of CSF and serum values in the blood–brain barrier. Correlations of serum and CSF values for free NAT concentration (A) or cell-bound NAT MFI (B). Correlations of CSF-to-serum ratios of albumin concentration (C) or IgG concentration (D) with the CSF-to-serum ratio of free NAT concentration. Values from NAT-treated patients with MS (black circle) who were partly reevaluated after 1 year (black square). *r* = Spearman’s rank correlation coefficient, *p* = *p* value of significance. (PPTX 270 kb)

## References

[1] Hutchinson M. Natalizumab: a new treatment for relapsing remitting multiple sclerosis. Ther Clin Risk Manag. 2007;3:259–68. 10.2147/tcrm.2007.3.2.259 PMC1936307 18360634

[2] Engelhardt B, Kappos L (2008). Natalizumab: targeting alpha4-integrins in multiple sclerosis. Neurodegener Dis.

[3] Clifford DB, DeLuca A, Simpson DM, Arendt G, Giovannoni G, Nath A (2010). Natalizumab-associated progressive multifocal leukoencephalopathy in patients with multiple sclerosis: lessons from 28 cases. Lancet Neurol.

[4] Bloomgren G, Richman S, Hotermans C, Subramanyam M, Goelz S, Natarajan A (2012). Risk of natalizumab-associated progressive multifocal leukoencephalopathy. N Engl J Med.

[5] Stüve O, Cravens PD, Frohman EM, Phillips JT, Remington GM, von Geldern G (2009). Immunologic, clinical, and radiologic status 14 months after cessation of natalizumab therapy. Neurology.

[6] Stüve O (2008). The effects of natalizumab on the innate and adaptive immune system in the central nervous system. J Neurol Sci.

[7] Khatri BO, Man S, Giovannoni G, Koo AP, Lee J-C, Tucky B (2009). Effect of plasma exchange in accelerating natalizumab clearance and restoring leukocyte function. Neurology.

[8] Wipfler P, Harrer A, Pilz G, Oppermann K, Afazel S, Haschke-Becher E, et al. Natalizumab saturation: biomarker for individual treatment holiday after natalizumab withdrawal? Acta Neurol Scand. 2013:129(3):e12-5. 10.1111/ane.12182 24032536

[9] Sheremata WA, Vollmer TL, Stone LA, Willmer-Hulme AJ, Koller M (1999). A safety and pharmacokinetic study of intravenous natalizumab in patients with MS. Neurology.

[10] Harrer A, Pilz G, Wipfler P, Oppermann K, Sellner J, Hitzl W, et al. High interindividual variability in the CD4/CD8 T cell ratio and natalizumab concentration levels in the cerebrospinal fluid of patients with multiple sclerosis. Clin Exp Immunol. 2015:180(3):383-92. 10.1111/cei.12590 PMC4449767 25603898

[11] Salhofer-Polanyi S, Baumgartner A, Kraus J, Maida E, Schmied M, Leutmezer F. What to expect after natalizumab cessation in a real-life setting. Acta Neurol Scand. 2014:130(2):97-102. 10.1111/ane.12250 24720783

[12] Prosperini L, Annovazzi P, Capobianco M, Capra R, Buttari F, Gasperini C, et al. Natalizumab discontinuation in patients with multiple sclerosis: profiling risk and benefits at therapeutic crossroads. Mult Scler. 2015;:1352458515570768. 10.1177/1352458515570768 25698174

[13] Tanaka M, Kinoshita M, Foley JF, Tanaka K, Kira J, Carroll WM (2015). Body weight-based natalizumab treatment in adult patients with multiple sclerosis. J Neurol Springer Berlin Heidelberg.

[14] Gerdes LA, Meinl I, Krumbholz M, Faber H, Weber F, Pellkofer HL, et al. De-escalation from natalizumab in multiple sclerosis: recurrence of disease activity despite switching to glatiramer acetate. J Neurol. 2011:258(9):1665-9. 10.1007/s00415-011-5996-y 21431380

[15] Ziemssen T, Kempcke R, Eulitz M, Großmann L, Suhrbier A, Thomas K (2013). Multiple sclerosis documentation system (MSDS): moving from documentation to management of MS patients. J Neural Transm.

[16] Shapiro RI, Plavina T, Schlain BR, Pepinsky RB, Garber EA, Jarpe M (2011). Development and validation of immunoassays to quantify the half-antibody exchange of an IgG4 antibody, natalizumab (Tysabri(®)) with endogenous IgG4. J Pharm Biomed Anal.

[17] Rispens T, Leeuwen AV, Vennegoor A, Killestein J, Aalberse RC, Wolbink GJ (2011). Measurement of serum levels of natalizumab, an immunoglobulin G4 therapeutic monoclonal antibody. Anal Biochem.

[18] Rispens T, Vennegoor A, Wolbink GJ, Polman CH, Killestein J. Natalizumab remains detectable in patients with multiple sclerosis long after treatment is stopped. Mult Scler. 2011:18(6):899-901. 10.1177/1352458511431073 22183929

[19] Benkert TF, Dietz L, Hartmann EM, Leich E, Rosenwald A, Serfling E (2012). Natalizumab exerts direct signaling capacity and supports a Pro-inflammatory phenotype in some patients with multiple sclerosis. Dieli F, editor. PLoS One.

[20] Börnsen L, Christensen JR, Ratzer R, Oturai AB, Sørensen PS, Søndergaard HB (2012). Effect of natalizumab on circulating CD4(+) T-cells in multiple sclerosis. PLoS One.

[21] Lindberg RLP, Achtnichts L, Hoffmann F, Kuhle J, Kappos L (2008). Natalizumab alters transcriptional expression profiles of blood cell subpopulations of multiple sclerosis patients. J Neuroimmunol.

[22] Wilson EH, Weninger W, Hunter CA (2010). Trafficking of immune cells in the central nervous system. J Clin Invest American Society for Clinical Investigation.

[23] Stüve O, Marra CM, Bar-Or A, Niino M, Cravens PD, Cepok S (2006). Altered CD4+/CD8+ T-cell ratios in cerebrospinal fluid of natalizumab-treated patients with multiple sclerosis. Arch Neurol.

[24] Schneider-Hohendorf T, Rossaint J, Mohan H, Böning D, Breuer J, Kuhlmann T (2014). VLA-4 blockade promotes differential routes into human CNS involving PSGL-1 rolling of T cells and MCAM-adhesion of TH17 cells. Journal of Experimental Medicine.

[25] Yu Y, Schürpf T, Springer TA (2013). How natalizumab binds and antagonizes α4 integrins. Journal of Biological Chemistry American Society for Biochemistry and Molecular Biology.

[26] Sechler JL, Cumiskey AM, Gazzola DM, Schwarzbauer JE (2000). A novel RGD-independent fibronectin assembly pathway initiated by alpha4beta1 integrin binding to the alternatively spliced V region. J Cell Sci.

[27] Hyun Y-M, Chung H-L, McGrath JL, Waugh RE, Kim M (2009). Activated integrin VLA-4 localizes to the lamellipodia and mediates T cell migration on VCAM-1. J Immunol.

[28] Rice GPA, Hartung H-P, Calabresi PA (2005). Anti-alpha4 integrin therapy for multiple sclerosis: mechanisms and rationale. Neurology.

[29] Nojima Y, Humphries MJ, Mould AP, Komoriya A, Yamada KM, Schlossman SF (1990). VLA-4 mediates CD3-dependent CD4+ T cell activation via the CS1 alternatively spliced domain of fibronectin. J Exp Med.

[30] Bomprezzi R, Pawate S (2014). Extended interval dosing of natalizumab: a two-center, 7-year experience. Ther Adv Neurol Disord.

[31] Zhovtis Ryerson L, Frohman TC, Foley J, Kister I, Weinstock-Guttman B, Tornatore C, et al. Extended interval dosing of natalizumab in multiple sclerosis. J Neurol Neurosurg Psychiatr. 2016;in press: doi:10.1136/jnnp-2015-312940. 10.1136/jnnp-2015-312940 26917698

[32] Fox RJ, Campbell Cree BA, de Seze J, Gold R, Hartung H-P, Jeffery D, et al. MS disease activity in RESTORE: a randomized 24-week natalizumab treatment interruption study. Neurology. 2014;82(17):1491-8. doi:10.1212/WNL.0000000000000355 10.1212/WNL.0000000000000355 PMC4011468 24682966

[33] Svenningsson A, Sundström P, Salzer J, Vågberg M, Fox RJ (2014). MS disease activity in RESTORE: a randomized 24-week natalizumab treatment interruption study. Neurology.

[34] Karceski S (2014). The RESTORE trial: what did we learn about multiple sclerosis?. Neurology.

[35] Hassoun L, Eisele J, Thomas K, Ziemssen T. Hands on Alemtuzumab-experience from clinical practice: whom and how to treat. Multiple Sclerosis and Demyelinating Disorders. 2016. in press. doi:10.1186/s40893-016-0011-1

[36] Pilz G, Harrer A, Oppermann K, Wipfler P, Golaszewski S, Afazel S (2012). Molecular evidence of transient therapeutic effectiveness of natalizumab despite high-titre neutralizing antibodies. Mult Scler.

[37] Vennegoor A, Rispens T, Strijbis EM, Seewann A, Uitdehaag BM, Balk LJ, et al. Clinical relevance of serum natalizumab concentration and anti-natalizumab antibodies in multiple sclerosis. Mult Scler. 2012:19(5):593-600. 10.1177/1352458512460604 22992450

[38] Haghikia A, Fischer M, Hellwig K, Linker R, Chan A, Hohlfeld R (2008). Open use of natalizumab. Neutralising antibodies and clinical data. Nervenarzt.

[39] Calabresi PA, Giovannoni G, Confavreux C, Galetta SL, Havrdova E, Hutchinson M (2007). The incidence and significance of anti-natalizumab antibodies: results from AFFIRM and SENTINEL. Neurology.

[40] Serana F, Chiarini M, Sottini A, Bertoli D, Giustini V, Vaglio Tessitore M (2014). Immunological biomarkers identifying natalizumab-treated multiple sclerosis patients at risk of progressive multifocal leukoencephalopathy. J Neuroimmunol.

