# Supplementary material for: New insights into the pharmacokinetics and pharmacodynamics of natalizumab treatment for patients with multiple sclerosis, obtained from clinical and in vitro studies
Source: J Neuroinflammation. 2016 Jun 27;13:164. doi: 10.1186/s12974-016-0635-2 (PMC4924246; doi:10.1186/s12974-016-0635-2)
Supplement: Additional file 1: Figure S1. — Standard curve of NAT assay. Specific numbers of HL60 cells were incubated with defined NAT concentrations and produced repeatable mean fluorescence intensity (MFI). These values were used to create a standard curve for NAT concentration (connected line). Specificity was tested using NAT-neutralising antibodies of a NAT-treated patient with multiple sclerosis (resulting value white circle). (PPTX 112 kb) [file 12974_2016_635_MOESM1_ESM.pptx]

## Slide 1
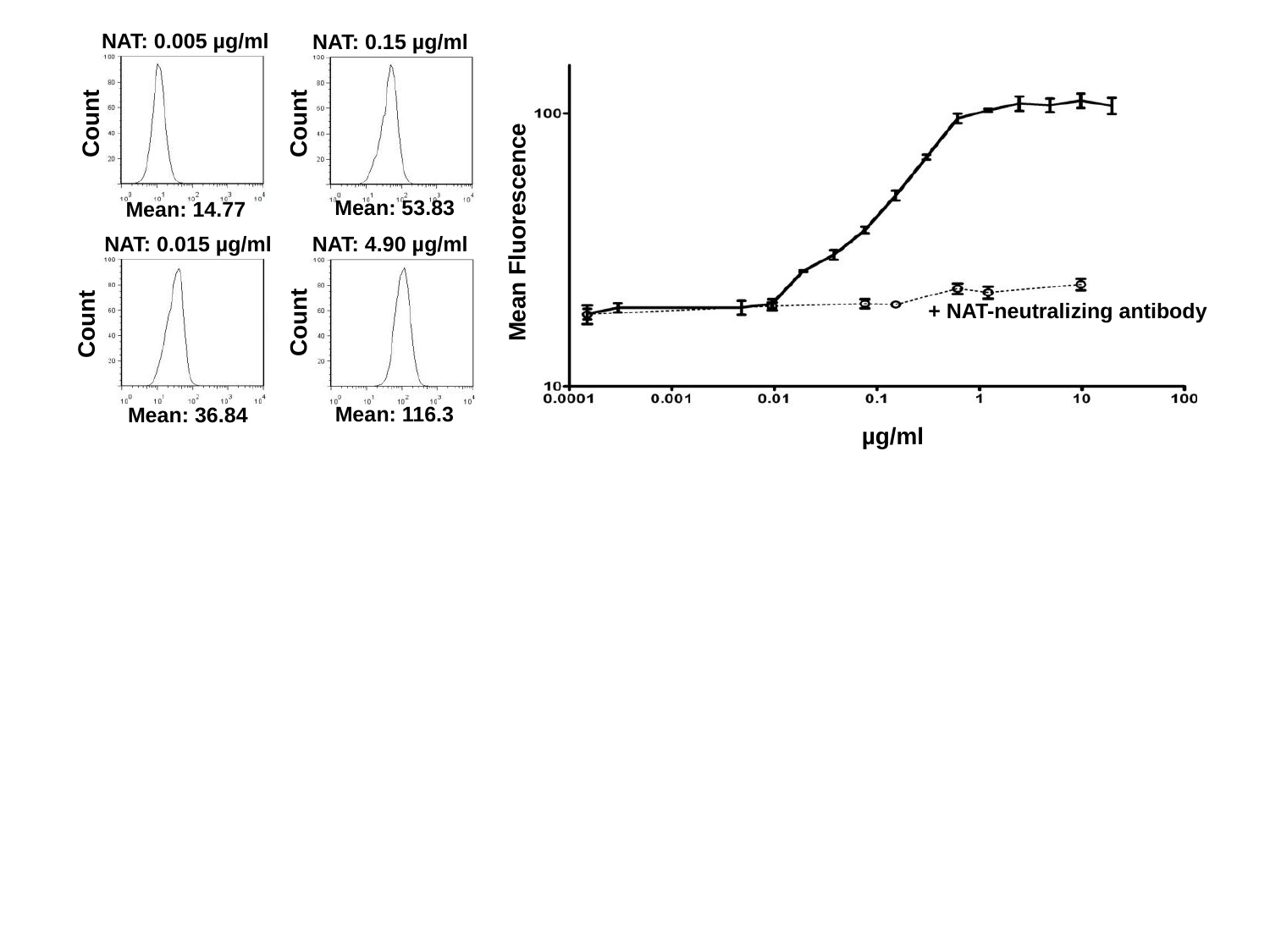

NAT: 0.005 µg/ml
NAT: 0.15 µg/ml
Count
Count
Mean Fluorescence
Mean: 53.83
Mean: 14.77
NAT: 0.015 µg/ml
NAT: 4.90 µg/ml
Count
Count
+ NAT-neutralizing antibody
µg/ml
Mean: 116.3
Mean: 36.84
