# Supplementary figures and images for: New insights into the pharmacokinetics and pharmacodynamics of natalizumab treatment for patients with multiple sclerosis, obtained from clinical and in vitro studies
Source: J Neuroinflammation. 2016 Jun 27;13:164. doi: 10.1186/s12974-016-0635-2 (PMC4924246; doi:10.1186/s12974-016-0635-2)

## Slide 1
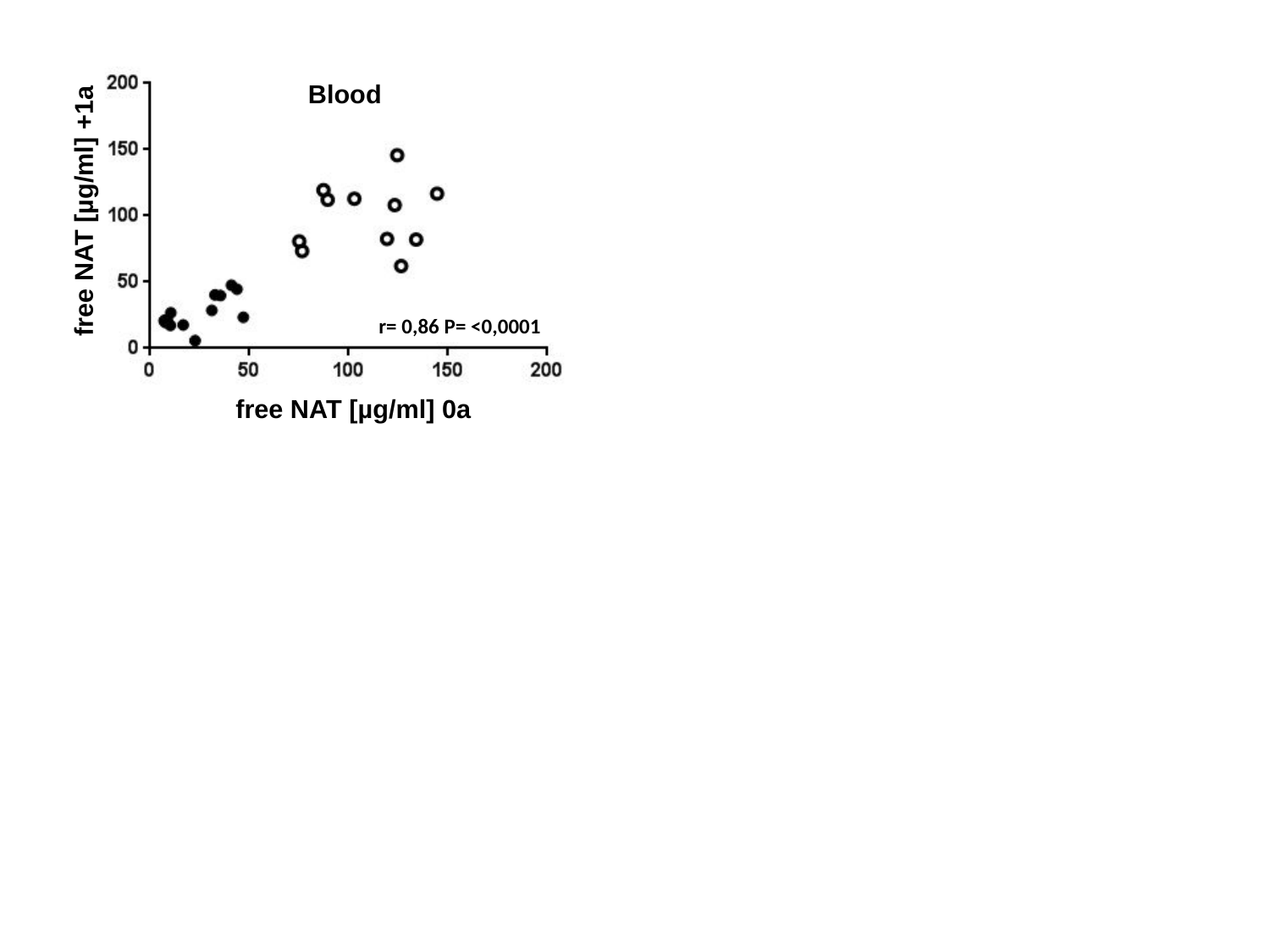

Blood
free NAT [µg/ml] +1a
r= 0,86 P= <0,0001
free NAT [µg/ml] 0a

Supplement: Additional file 2: Figure S3. — Correlation of pre- and post-serum values at both timepoints. Correlation of pre- (black circle) and post- (white circle) NAT serum values at timepoint 0 and after 1 year (+1a). r = Spearman’s rank correlation coefficient, p = p value of significance. (PPTX 45 kb) [file 12974_2016_635_MOESM2_ESM.pptx]
