# Supplementary material for: New insights into the pharmacokinetics and pharmacodynamics of natalizumab treatment for patients with multiple sclerosis, obtained from clinical and in vitro studies
Source: J Neuroinflammation. 2016 Jun 27;13:164. doi: 10.1186/s12974-016-0635-2 (PMC4924246; doi:10.1186/s12974-016-0635-2)
Supplement: Additional file 3: Figure S2. — Correlation of CSF and serum values in the blood–brain barrier. Correlations of serum and CSF values for free NAT concentration (A) or cell-bound NAT MFI (B). Correlations of CSF-to-serum ratios of albumin concentration (C) or IgG concentration (D) with the CSF-to-serum ratio of free NAT concentration. Values from NAT-treated patients with MS (black circle) who were partly reevaluated after 1 year (black square). r = Spearman’s rank correlation coefficient, p = p value of significance. (PPTX 270 kb) [file 12974_2016_635_MOESM3_ESM.pptx]

## Slide 1
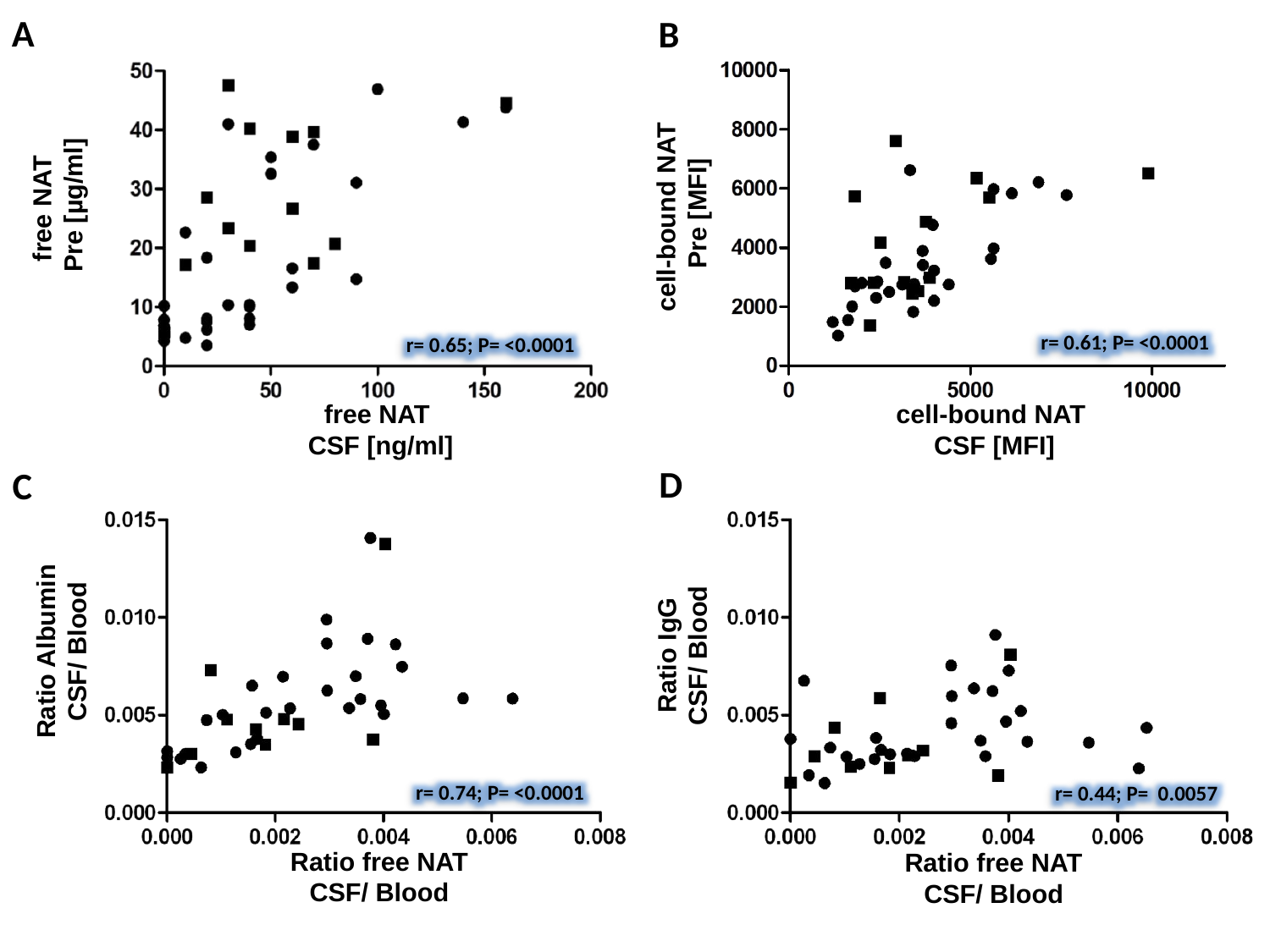

A
B
free NAT
Pre [µg/ml]
r= 0.65; P= <0.0001
free NAT
CSF [ng/ml]
cell-bound NAT
Pre [MFI]
r= 0.61; P= <0.0001
cell-bound NAT
CSF [MFI]
D
C
Ratio IgG
CSF/ Blood
r= 0.44; P= 0.0057
Ratio free NAT
CSF/ Blood
Ratio Albumin
CSF/ Blood
r= 0.74; P= <0.0001
Ratio free NAT
CSF/ Blood
